# Minimally Invasive Surgery for Spontaneous Intracerebral Hematoma. Real-Life Implementation Model and Economic Estimation

**DOI:** 10.3389/fneur.2022.884157

**Published:** 2022-05-02

**Authors:** Alejandra Mosteiro, Sergi Amaro, Ramon Torné, Leire Pedrosa, Jhon Hoyos, Laura Llull, Luis Reyes, Abel Ferrés, Nicolás de Riva, Ricard Mellado, Joaquim Enseñat

**Affiliations:** ^1^Department of Neurosurgery, Hospital Clinic, University of Barcelona, Barcelona, Spain; ^2^Comprehensive Stroke Center, Barcelona, Spain; ^3^Department of Neurology, Hospital Clinic, University of Barcelona and August Pi I Sunyer Biomedical Research Institute (IDIBAPS), Barcelona, Spain; ^4^IDIBAPS Biomedical Research Institute, Barcelona, Spain; ^5^Department of Anesthesiology, Hospital Clinic de Barcelona, University of Barcelona, Barcelona, Spain; ^6^Department of Anesthesiology and Critical Care, Hospital Clinic de Barcelona, University of Barcelona, Barcelona, Spain

**Keywords:** hemorrhagic stroke, spontaneous cerebral hematoma, minimally invasive surgery, evacuation, cost-utility

## Abstract

**Objective:**

Spontaneous intracerebral hemorrhage is characterized by high fatality outcomes, even under best medical treatment. Recently, minimally invasive surgical (MIS) evacuation of the hematoma has shown promising results and may soon be implemented in the clinical practice. Hereby, we intended to foresee the logistic requirements for an early hematoma evacuation protocol, as well as to evaluate in a real-life implementation model the cost-utility of the two main MIS techniques for hemorrhagic stroke (catheter evacuation plus thrombolysis and neuroendoscopic aspiration).

**Methods:**

Data were obtained from the pool of hemorrhagic-stroke patients admitted to our institution during an annual period (2020–2021) and contrasted to the reported results in published trials of MIS techniques. Potential candidates for surgical treatment were identified according to the inclusion/exclusion criteria established in these trials. Then, a cost-utility analysis was performed, which explored the incremental cost per unit of health gained with a given treatment. The treatment effect was measured by differences in modified Rankin Score, and subsequently converted to quality-adjusted life years (QALY).

**Results:**

Of the 137 patients admitted to our center with supratentorial spontaneous intracerebral hemorrhage in a 1-year period, 17 (12.4%) were potential candidates for the catheter evacuation plus thrombolysis technique (Minimally Invasive Surgery with Thrombolysis in Intracerebral Hemorrhage Evacuation trial, MISTIE III criteria) and 59 (43.0%) for the neuroendoscopic aspiration technique (Dutch Intracerebral Hemorrhage Surgery Trial Pilot Study, DIST criteria). The incremental cost-utility ratio was € 76,533.13 per QALY for the catheter-based evacuation and € 60,703.89 per QALY for the endoscopic-based technique.

**Conclusion:**

Around 12–43% of patients admitted to hospital with spontaneous hemorrhagic stroke could be potential candidates to MIS early evacuation of the cerebral hematoma. In our real-life implementation model, the cost-utility analysis favored the neuroendoscopic evacuation over the catheter aspiration technique. Further studies are advisable as new data from the ongoing randomized trials becomes available.

## Introduction

Spontaneous intracerebral hemorrhage (ICH) affects 10–22 per 100,000 person-years, a figure estimated to increase as the population ages and the use of antithrombotics becomes more extended (1). This high incidence is aggravated by inherent fatality outcomes, by which half of these patients are dead at 1 year and only 12–39% of the survivors achieve long-term functional independence (1). Yet, the management of ICH is still a challenge for modern medicine since no specific treatment exists. In this context, conservative measures are currently the mainstream for primary ICH, including coagulopathy reversal, blood pressure and glucose control, and seizure treatment. Meanwhile, surgical intervention is reserved for life-threatening situations, such as invasive intracranial pressure monitoring in critical comatose patients, placement of an external ventricular drainage for acute hydrocephalus, hematoma evacuation in deteriorating patients, and decompressive craniectomy in comatose patients with significant midline shift or refractory intracranial hypertension (2, 3).

Recently, several randomized controlled trials have evaluated the potential benefits of early surgical evacuation of ICH (4–7). Special interest has been arisen by minimally disruptive techniques, with two main variants that include passive hematoma evacuation through a catheter plus inoculation of a thrombolytic agent and active aspiration under direct endoscopic view (8). Although no conclusive indication for prompt evacuation has been settled, minimally invasive surgery (MIS) has proven to reduce mortality in deep-seated primary hematomas. Furthermore, *post hoc* analysis have shown that MIS could reduce the neurologic sequelae, provided an optimal neurosurgical performance is achieved and appropriate patient selection is accomplished (9–14).

Thus, MIS intervention is expected to accelerate the functional recovery and, therefore, to minimize hospital stay and maximize resumption of an active lifestyle. However, MIS techniques, regardless of the variant, entail higher direct medical costs compared to the prevailing conservative alternative. Still, the feasibility of MIS in the routine practice and its viability in terms of expenses has not been adequately addressed (15). In the plausible scenario in which MIS becomes part of the standardized treatment of ICH, practitioners should be aware not only of the technical aspects of the procedure itself, but also of the impact that its implementation will have on hospital dynamics and health funds.

For this matter, we employed real-life data from the pool of ICH patients admitted to our institution during an annual period (2020–2021) and contrasted it to the reported results of two MIS techniques: MISTIE III (Minimally Invasive Surgery with Thrombolysis in Intracerebral Hemorrhage Evacuation III) randomized trial, as the paradigm of catheter plus thrombolysis, and Kellner et al. (11) trial (11), as the most complete available study of neuroendoscopic evacuation. Our preliminary hypothesis is that a MIS protocol could be cost-effective, when compared to conservative treatment, in patients with spontaneous ICH who meet the inclusion/exclusion criteria of the clinical trials of reference.

## Materials and Methods

### Population of Reference

The patients included in this retrospective analysis were part of a prospectively collected clinical registry of patients with spontaneous ICH admitted in our referral comprehensive stroke center, between March 2020 and March 2021. Ours is a third-level neurosurgical center in a European middle-income country, which serves as a community hospital to a population of 540,000 inhabitants and provides tertiary acute stroke care to a population of 2,200,000 inhabitants. The specific selection criteria for this analysis were: (1) admission within the first 48 h after supratentorial spontaneous ICH onset and (2) absence of relevant premorbid clinical disability [modified Rankin Scale score (mRS) lower than or equal to 3]. First, we extracted the number of patients-per-year that would had been operated in our institution if a MIS protocol for ICH had been implemented. To this aim, we applied the inclusion/exclusion criteria of the main clinical trials on the subject and obtained the number of patients that would potentially have been treated with MIS in our hospital. The clinical trials included were: MISTIE III (Minimally Invasive Surgery with Thrombolysis in Intracerebral Hemorrhage Evacuation III), INVEST (Minimally Invasive Endoscopic Surgery with Apollo in patients with Brain Hemorrhage), DIST (Dutch Intracerebral Hemorrhage Surgery Trial Pilot Study) and ENRICH (Early MiNimally-invasive Removal of IntraCerebral Hemorrhage) (Supplementary Table S1) (4–7). In addition, we collected relevant data for functional and economic evaluation: epidemiological information, Glasgow Coma Scale (GCS) and National Institute of Health Stroke Scale (NIHSS) upon arrival, time between triage and neuroimaging, hematoma volume and location, hematoma stability/expansion, number of patients who underwent conventional rescue surgery, duration of surgery, number of external ventricular drains placed, length of stay (LoS) in both the intensive care unit (ICU) and the Stroke Unit, total duration of hospital admission, mRS and NIHSS at discharge, discharge location, and mRS at 3 months. Midterm evaluation of functional dependence (according to mRS) was done by trained clinicians 3 months after the event, as part of the clinical routine in our center. Hematoma volume was calculated using the ABC/2 formula (16), both on the initial and control neuroimaging (either CT or MRI).

### Surgical Technique and Outcome Measurement

Many variants of the MIS technique for hematoma evacuation have been proposed, each one with particular requirements regarding infrastructure, surgical material, technology and human resources. For the current analysis, we used the most complete and up-to-date published data representing the two main surgical variants, as follows. To evaluate the catheter evacuation prototype, we obtained data from the MISTIE III randomized trial (4). Meanwhile, to evaluate the mechanical evacuation, we obtained data from the comparative study performed by Kellner et al. (11), since no data from randomized trials were available at the moment of this analysis. In particular, we used the reported data related to mean surgical duration, ICU and hospital stay, discharge location, mortality at 30 days and midterm functional status (mRS at 180 days in Kellner and mRS at 365 days in MISTIE III).

Data were obtained from three different sources. First, data of the conservative cohort belong to a retrospective review of a prospectively collected database containing all patients admitted to our institution with primary supratentorial ICH during the annual period of 2020–2021. Second, data of the interventional cohort treated with catheter evacuation were extracted from the published results of a prospective randomized clinical trial (MISTIE III). Third, data of the interventional cohort treated with neuroendoscopic evacuation were retrieved from the published results of a retrospective single-center study (11). The groups of comparison for each of the techniques were established as follows. In the catheter-based technique, the control group consisted of the patients from our institution who were potential candidates for MISTIE III, based on the inclusion and exclusion criteria of the original study protocol; meanwhile, the treatment group was the experimental cohort from the randomized trial. In the neuroendoscopic procedure, the control group included the patients from our institution who were potential candidates for DIST, based on the inclusion and exclusion criteria of the study protocol; while the treatment group was the experimental cohort in Kellner et al. trial (11).

MISTIE III was a phase III randomized blinded controlled trial evaluating the benefits of catheter evacuation plus rTPA administration for ICH patients. The interventional group underwent image-guided insertion of a catheter into the long axis of the hematoma; afterwards, soft hematoma aspiration was done until resistance was met. Then, the catheter was tunneled subcutaneously, the patient transferred to the ICU. If postoperative CT scan confirmed appropriate catheter placement, sequential doses of alteplase were administered through the catheter. Time window for the intervention was 72 h. The primary endpoint was a mRS score of 0–3 at 1 year. The trial did not demonstrate better rates of good outcome in the interventional group; however, it showed benefits in terms of mortality and a potential benefit in the subgroup of patients with a residual hematoma of ≤ 15 ml.

On the other hand, DIST is a currently ongoing, single-masked, multicenter phase II trial. In this case, the intervention consists of endoscopy-guided surgery within 8 h of symptom onset. Primary endpoints are safety and technical effectiveness; meanwhile, functional outcome (mRS on days 90 and 180) is the secondary outcome. As it follows, both MISTIE III and DIST aimed to assess functional outcomes but in an unequal way (primary vs. secondary objective and at different time points). A power analysis was conducted in MISTIE III to determine the sample size (250 patients in each arm for an 88% power at α level 0.05 to detect an average effect size of 13%). As a matter of fact, methodology and recruitment are more robust in MISTIE III. Since the results from DIST are not available at the present moment, no formal comparison between the outcomes achieved in the two studies can be done.

Finally, Kellner 2020 trial was a single center retrospective study of prospectively collected data evaluating the long-term functional outcomes from a cohort of neuroendoscopically treated ICH patients. The primary outcome was the proportion of patients with mRS 0–3 at 6 months. According to the authors, “favorable long-term functional outcome, defined as mRS 0–3, was observed in 46 of the 100 included patients. The mortality rate at discharge, 30 days, and 180 days was 3%, 9% and 16%, respectively”.

In the neuroendoscopic procedure, was the experimental cohort in Kellner et al. trial (11). Data could not be extracted from an experimental cohort belonging to a controlled trial of neuroendoscopic evacuation because, at the moment of writing these lines, such a trial is not available in the literature. To overcome this limitation, we opted for Kellner et al. (11) study, since it offered detailed information about logistics and outcomes, even when it was not a randomized study.

Clinical outcomes were defined according to the pre-established clinical endpoints of both mentioned studies: mRS at 1 year and mortality at 30 days, in MISTIE III; mRS at 180 days and mortality at 30 days, in Kellner et al. For outcome quantification, the unit of health benefit used was Quality-adjusted life years (QALY), because it is the most widely used unit in economic estimations and it facilitates the comparison to other health strategies. QALY were obtained from mRS scores based on the model from Whynes et al. (17); length of life was assumed to be 1 year, as this was the longest follow-up period in the studied cohorts. Patients who did not survive until the midterm evaluation were attributed 0 QALY. Supplementary Table 2 provides a scope view of the strategy. The transformation was thus obtained by multiplying the number of years of survival (i.e., one) by the mRS achieved after midterm recovery.

### Cost Estimation

General costs were obtained from the public health tariff 2020/2021 of our institution, which is part of the national welfare system. Basic surgical costs, including operating room (OR), instruments, consumables and neuronavigation systems were also obtained from our hospital health tariff (18). To estimate the surgical costs of the novel MIS techniques, we added up the specific materials to the standard OR costs. We registered every resource used per patient based on their medical records and then computed the unit cost of each resource to obtain a global mean cost per patient and per intervention. In the conservative treatment group, to account for rescue surgery, a probability of emergency intervention was calculated based on our institutional cohort data. Then, this probability was multiplied by the average cost of the rescue procedure. Further on, we included indirect medical costs as costs derived from discharge to a secondary rehabilitation or social facility; in these cases, costs were based on publicly published data (19).

### Economic Analysis

Economic evaluation consisted of a cost-utility analysis where the two treatment strategies were confronted, namely the standard medical treatment against each of the proposed MIS interventions. The same method was applied separately for the catheter-based and the endoscopic-based procedures. The cost-utility equation explored the incremental cost per unit of health gained with a given treatment. Costs were expressed as mean cost per patient. Then, mean incremental cost and mean incremental effectiveness were calculated for each treatment group. The cost-effectiveness ratio (ICER) was defined as the ratio between the incremental cost and the incremental effectiveness of the two intervention alternatives, as follows:


$$ICER\;=\;\frac{Cost\;of\;MIS-Cost\;of\;Conventional\;Treatment}{QALY\;MIS-QALY\;Conventional\;Treatment}$$

For graphical purposes, ICER values of the two MIS variants were represented in a cost-utility plane, which consists of four quadrants. The north-east corner indicates a more expensive and more effective intervention; the south-east corner contains less costly but more effective intervention; the north-west corner stands for costlier yet less effective intervention; the south-west corner is a less expensive and less effective intervention. Cost-utility acceptability curves were used to represent the uncertainty concerning the cost-utility of each intervention, as an alternative to confidence intervals around ICER. The curves represent the probability that a particular intervention is optimal, over a range of willingness-to pay thresholds (20). Additionally, the net health benefit was calculated upon a theoretical willingness-to-pay threshold of € 30,000 per QALY, which is the most widely accepted cut-off point in our setting (21).

Calculations were performed using Microsoft Excel XP^TM^ and SPSS (IBM version 23.0) and graphics were obtained with R Studio version 1.4.1103. The present analysis followed the Health Economic Evaluation Reporting Standards (CHEERS) guidelines (22) for communicating economic evaluations of health interventions. No statistical tests were conducted as neither hypothesis testing nor the level of statistical significance were relevant to our analysis.

### Ethical Statement

The study protocol was approved by the Clinical Research Ethics Committee from the participating institution (Reg. HCB/2021/1282) and complies with national legislation in the field of biomedical research, the protection of personal data (15/1999) and the standards of Good Clinical Practice, as well as with the Helsinki Declaration (1975 and 1983 revisions). Patient records were anonymized before analysis.

## Results

### Real-Life Cohort of ICH and Potential Candidates to MIS

One hundred thirty-seven patients were admitted to our tertiary hospital suffering from supratentorial spontaneous ICH during a 1-year period (2020–2021). Demographic and clinical data are shown in Table 1. Of those 137 patients, the number of patients who met the inclusion and exclusion criteria established across the different surgical randomized trials was 17 (12.4%) for the MISTIE III trial, 59 (43.0%) for the DIST trial, 24 (17.5%) for the ENRICH trial; and 11 (8.0%) for the INVEST trial. The specific inclusion and exclusion criteria of these trials are detailed in Supplementary Table S1.

**Table 1:** Baseline clinical and radiological features of the institutional sample.

	**Global** ***N***** = 137**	**MISTIE III candidates** ***N***** = 17**	**DIST candidates** ***N***** = 59**
Age (years), mean (SD)	69 (15.1)	73 (12.3)	71 (13.7)
Sex (male)	81 (59.1)	12 (70.6)	38 (64.4)
Previous antithrombotic			
Oral anticoagulant	21 (15.3)	0 (0)	9 (15.3)
Heparin	3 (2.2)	0 (0)	1 (1.7)
Antiplatelets	29 (19.0)	6 (35.3)	12 (20.3)
Combinations	2 (1.5)	1 (5.9)	1 (1.7)
Premorbid mRS			
0	90 (65.7)	15 (88.2)	44 (74.6)
1	14 (10.2)	2 (11.8)	6 (10.2)
2	18 (13.1)	0 (0)	9 (15.3)
3	15 (11.0)	0 (0)	0 (0)
GCS			
14–15	76 (55.5)	7 (41.1)	34 (57.6)
5–13	47 (34.3)	10 (58.9)	25 (42.4)
3–4	14 (10.2)	0 (0)	0 (0)
NIHSS, median (range)	12 (0–36)	18 (6–27)	12 (0–30)
Location			
Basal ganglia	73 (53.3)	7 (41.2)	32 (54.2)
Subcortical	15 (10.9)	1 (5.0)	3 (5.1)
Cortical	46 (33.6)	9 (52.9)	24 (40.7)
Brainstem	3 (2.2)	0 (0)	0 (0)
IVH	52 (38.0)	5 (29.4)	23 (39.0)
Hematoma volume (ml), mean (SD)	35 (42.0)	60 (36.3)	40 (38.3)
Hematoma expansion (>5 ml)	46 (33.6)	0 (0)	20 (33.9)

*The first column includes all patients admitted to hospital with the diagnosis of spontaneous intracerebral hemorrhage. The second and third columns refer to patients who met MISTIE III and DIST inclusion/exclusion criteria*.

*Data are n (%) unless otherwise specified. GCS, Glasgow Coma Scale score; IVH, Intraventricular Hemorrhage; mRS, modified Rankin Scale; NIHSS, National institute of Health Stroke Scale; SD, Standard Deviation*.

The median time between triage to first neuroimaging (head CT scan) was 19 (2–460) min. Most cases had a control image within the first 24 h (93 out of 137, 67.9%). In this subset, 33.6% had an enlarged hematoma defined by an increase in volume of more than 5 ml. Surgery was performed in 13.1% of the studied patients because of neurological deterioration or low GCS at admission due to mass effect (Table 2). The intervention consisted of craniotomy and hematoma evacuation under microscopic vision.

**Table 2:** Data on clinical management, resources utilized and outcomes in the institutional cohort.

	**Global** ***N***** = 137**	**MISTIE III candidates** ***N***** = 17**	**DIST candidates** ***N***** = 59**
Time to image (min)			
Mean (SD)	52 (89.8)	34 (66.1)	47 (75.1)
Median (range)	19 (2–460)	17 (9–290)	15 (6–136)
Surgery performed	18 (13.1)	5 (29.4)	10 (16.9)
EVD	20 (14.6)	4 (23.5)	12 (20.3)
Time to surgery (min)			
Mean (SD)	164 (139.1)	103 (43.9)	111 (48.6)
Median (range)	120 (60–600)	90 (75–180)	86 (60–196)
GCS pre-surgery, mean (SD)	9 (4)	9 (3)	10 (3)
LoS in ICU (days), mean (SD)	2 (7.8)	5 (8.8)	4 (11.5)
LoS in Stroke Unit (days), mean (SD)	2 (2.6)	2 (2.6)	3 (2.8)
LoS in hospital (days), mean (SD)	15 (20.8)	34 (42.6)	23 (27.3)
Discharge location:			
Home	39 (28.5)	*0*	12 (20.3)
Rehabilitation	54 (39.4)	12 (70.6)	33 (55.9)
Death	44 (32.1)	5 (29.4)	13 (22.0)
Survival (days), mean (SD)	186 (182.8)	217 (184.3)	447 (193.4)
mRS at discharge			
0	4 (2.9)	*0*	1 (1.7)
1	11 (8)	*0*	5 (8.5)
2	22 (16.1)	2 (11.8)	7 (11.9)
3	12 (8.8)	*0*	5 (8.5)
4	23 (16.8)	6 (35.3)	12 (20.3)
5	20 (14.6)	4 (23.5)	16 (27.1)
6	44 (32.1)	5 (29.4)	13 (22)
NIHSS at discharge, mean (SD)	6.9 (6.6)	12.9 (5.9)	8.7 (7.2)

*Data are n (%) unless otherwise specified. EVD, External Ventricular Drain; GCS, Glasgow Coma Scale score; LoS, Length of Stay; ICU, Intensive care Unit; mRS, modified Rankin Scale; NIHSS, National institute of Health Stroke Scale; SD, Standard Deviation*.

### Acute and Midterm Outcomes of Potential MIS Candidates

Upon admission, patients were transferred to either the ICU or the Stroke Unit. Mean length of stay at these units for MISTIE III potential candidates was 5 (± 8.8) days and 2 (± 2.6) days, respectively. Meanwhile, the length of stay for DIST potential candidates was 4 (± 11.5) days in the former unit and 3 (± 2.8) days in the latter unit. The total hospital stay was 34 (± 42.6) days for MISTIE III candidates and 23 (± 27.3) for DIST candidates. Only 20.3% of DIST candidates, and none of the MISTIE III candidates, were able to return home at hospital discharge. Five (29.4%) of MISTIE III-like and 13 (22.0%) of the DIST-like patients died in the acute phase. Of those who survived, 2 (11.8%) and 18 (30.6%), respectively, had a moderately good functional status at discharge (mRS ≤ 3), while 2 (14.3%) and 14 (28.6%) did so at 3 months (Table 2).

### Economic Estimate of the Catheter-Based Evacuation

In the subgroup of patients from our institution who could have been candidates for catheter evacuation, the total cost of the conventional treatment provided at our center was € 14,190.06 per patient (Table 3). If social costs were to be taken into account–with an estimation of a 3-month stay in a rehabilitation suite for those not able to return home at discharge–the total amount would have ascended to € 20,406.18 (Table 3). The inferred total cost of treatment with the catheter-based procedure, according to the data of the MISTIE III trial, was € 33,127.15 per patient (Table 3). Thus, the ICER of the catheter intervention was € 82,335.17 per QALY (Table 4).


$$ICER_{catheter}\;=\;\frac{33,127.15-14,190.06}{0.32-0.09}\;=\;€\;82,335.17\;per\;QALY$$

**Table 3:** Comparison of hospital resources needed for the two treatment alternatives and estimation of total costs.

	**MISTIE III candidates** ***N*** **= 17**	**MISTIE III** ***N*** **= 255**
Ictus to randomization (minutes), median (range)	17 (9–290)	47 (33–60)
Surgery duration (hours), median (range)	2.1 (0.8–5)	1 (1–1)
LoS in ICU (days), median (range)	4 (0–32)	10 (7–17)
LoS in Stroke Unit (days), median (range)	2 (0–7)	-
LoS in hospital (days), median (range)	24 (1–174)	55 (34–105)
Mortality at 30 days	4 (23.5)	24 (9)
mRS midterm	Data available from 14 patients	
0	0	1 (<1)
1	0	15 (6)
2	0	30 (12)
3	2 (14.3)	64 (26)
4	2 (14.3)	60 (24)
5	3 (21.4)	31 (12)
6	7 (50)	48 (19)
Cost per intervention (€)		
Operating room	€ 1,800 ^*^ 29% = € 529.2	€ 2,512
Price per minute	130 min ^*^ € 5/min = € 650	60 min ^*^ € 5/min = € 300
Surgical pack	€ 1,150	€ 1,150
Navigation system	0	€ 862
Specific material	0	€ 200
ICU	4 days ^*^ €1,175.9/day = € 4,703.5	10 days ^*^ €1,175.9/day = € 11,758.8
Stroke Unit	2 day ^*^ €707.4/day = € 1,414.8	0
Hospitalization	18 days ^*^ €419/day = € 7,542.54	45 day ^*^ €419/day = € 18,856.4
Social/rehabilitation facility	70.6% ^*^ 97.8 €/day ^*^ 90 days = € 6,216.1	Not reported

*The first column refers to the conservative treatment offered to patients from the institution who were potential candidates for MISTIE III. The second column refers to the catheter-based evacuation treatment given to the intervention group in the MISTIE III trial*.

*Data are n (%) unless otherwise specified. LoS, Length of Stay; ICU, Intensive care Unit; mRS, modified Rankin Scale*.

* *, Multiplication mathematic symbol*.

**Table 4:** Cost-utility analysis of minimally invasive surgical techniques in hemorrhagic stroke; the reference is conventional management.

	**Conventional**	**Catheter**	**Endoscopic**
Cost (€)	14,190.1–13,486.5^*^	33,127.2	23,318.7
Incremental cost (€)		18,937.1	9,832.2
Effectiveness measure:			
mRS ≤ 3 at midterm	14.3–16.3%^*^	45%	46%
Survival at 30 days	76.3–76.5%^*^	91%	91%
QALY	0.1–0.2^*^	0.3	0.4
ICER (€ per QALY)		82,335.2	75,047.4

*Total costs are based on data from Tables 3, 5*.

*ICER, Incremental Cost-Effectiveness Ratio*.

* *The first value corresponds to the catheter control group of potential candidates for MIS, and the second value, to the endoscopic control group*.

**Figure 1:**
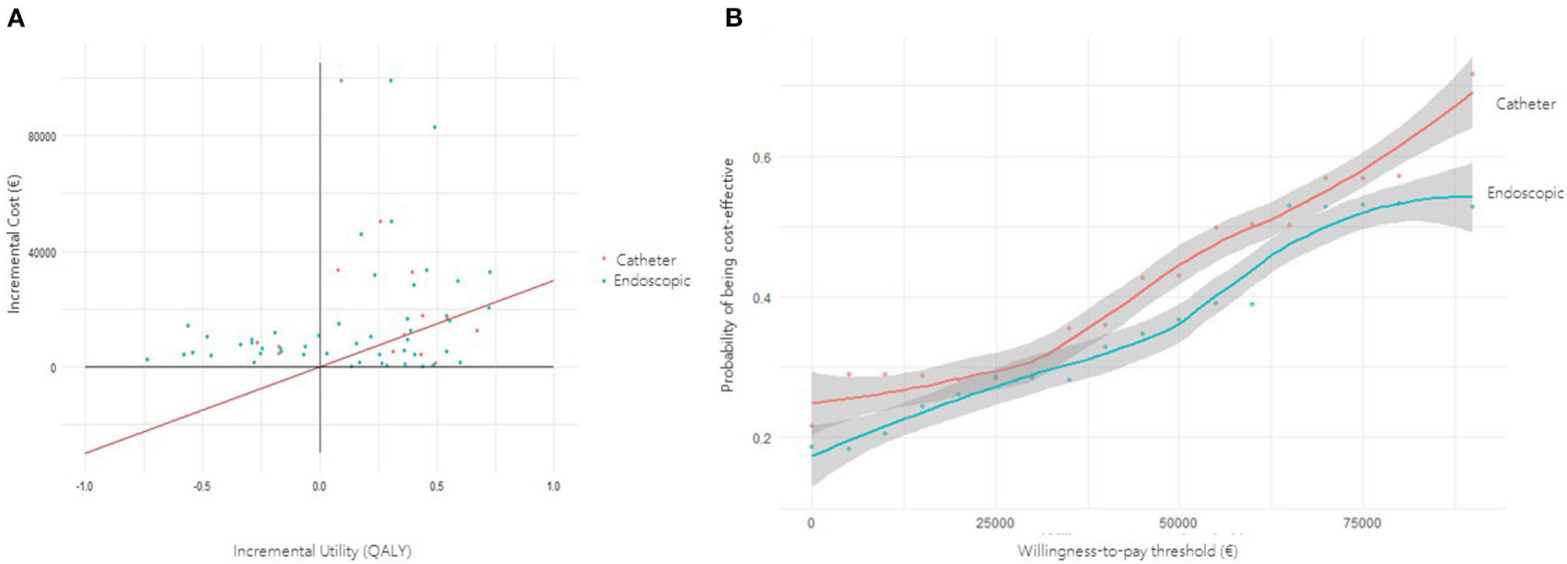
**(A)** Incremental cost-utility scatter plot. Each point represents a simulation of a real-life case treated with minimally invasive (MIS) hematoma evacuation. Catheter-based replicates are represented in red, whereas endoscopic replicates are in blue. The diagonal red line represents the willingness-to-pay threshold of € 30,000 per QALY; points situated to the right of this theoretical threshold are considered cost-effective. **(B)** Cost-utility acceptability curve. The curve presents the probability of MIS being cost-effective at any given willingness-to-pay threshold.

In the cost-utility graphic (Figure 1), the majority of the replicates fell in the north-east corner, which indicates a costlier and more effective intervention, when compared to the conventional assistance. Only two of the replicates fell in the north-west corner, indicating a costlier but less effective intervention.

### Economic Estimate of the Neuroendoscopic Evacuation

Similarly, we analyzed the cohort of patients from our institution who could have been candidates for endoscopic evacuation of the hematoma. In this cohort, the total cost per patient for the conventional treatment was € 13,486.53. Given that 55.9% of patients were discharged to a rehabilitation facility, the global cost including indirect social costs within the first 3 months would be € 18,408.36 per patient (Table 4). The inferred total cost if those patients had been treated with the endoscopic intervention, according to the data from Kellner et al. (11), would have been € 23,318.74 per patient. In Kellner trial, 93% of patients were discharged to a secondary institution; with this social cost in mind, the global cost per patient during the first 3 months would have been € 31,507.11 (Table 5). The ICER of the endoscopic-based strategy was € 75,047.40 per QALY (Table 4).


$$ICER_{endoscopic}\;=\;\frac{23,318.74-13,486.53}{0.365-0.234}\;=\;€\;75,047.40\;per\;QALY$$

**Table 5:** Comparison of hospital resources needed for the two treatment alternatives, plus estimation of total costs.

	**DIST candidates** ***N***** = 59**	**KELLNER 2020** ***N***** = 100**
Ictus to randomization (minutes), mean (SD)	47 (75.1)	38 (28.7)
Surgery duration (hours), mean (SD)	2 (1.2)	3 (1.1)
LoS in ICU (days), median (range)	4 (0–18)	9 (5–14)
LoS in Stroke Unit (days), median (range)	3 (0–6)	-
LoS in hospital (days), median (range)	23 (0–34)	17 (9–24)
Discharge location		
Home	12 (20.3)	4
Rehabilitation	33 (55.9)	93
Death	13 (22)	3
Mortality at 30 days	14 (23.7)	9
mRS midterm	Data available from 49 patients	
0	2 (4.1)	1
1	1 (2)	9
2	5 (10.2)	19
3	6 (12.2)	17
4	10 (20.4)	25
5	6 (12.2)	13
6	19 (38.7)	16
Cost per intervention (€)		
Operating room	€1,834.5 ^*^ 16.9% = € 310.03	€ 9,762
Price per minute	136.9 min ^*^ €5/min = € 684.5	150 min ^*^ €5/min = € 750
Surgical pack	€ 1,150	€ 1,150
Navigation system	0	€ 862
Specific material	0	€ 7,000
ICU	4 days ^*^ €1,175.9/day = € 4,703.5	8.5 days ^*^ €1,175.9/day = € 9,995
Stroke unit	2.5 days ^*^ €707.4/day = € 1,768.50	0
Hospitalization	16 days ^*^ €419/day = € 6,704.5	8.5 days ^*^ €419/day = € 3,561.8
Social/rehabilitation facility	55.9% ^*^ € 97.8/day ^*^ 90 days = € 4,921.8	93% ^*^ €97.8/day ^*^ 90 days = € 8,188.4

*The first column refers to the conservative treatment offered to patients from the institution who were potential candidates for DIST. The second column refers to the endoscopic-based evacuation given to the intervention group in Kellner et al. trial*.

*Data are n (%) unless otherwise specified. LoS, Length of Stay; ICU, Intensive care Unit; mRS, modified Rankin Scale; SD, Standard Deviation*.

* *, Multiplication mathematic symbol*.

In the cost-utility graphic (Figure 1), the majority of the replicates fell in the north-east corner, thus indicating a costlier and more effective intervention.

In order to meet the cost-effectiveness threshold of € 30,000 per QALY, the effect size that MIS would need to achieve is 0.72 QALY in the catheter intervention and 0.56 QALY in the endoscopic intervention.

### Cost-Utility Acceptability Curve

In the cost-utility plane (Figure 1), both MIS techniques showed a trend toward a more expensive and more effective intervention than the conventional alternative. Most of the replicated were upon the € 30,000 willingness-to-pay threshold line. According to the cost-utility acceptability curve, to obtain a 50% probability of cost-utility, a willingness-to-pay threshold of € 62,000 would be needed for the catheter technique and € 69,000 for the endoscopic technique (Figure 1).

## Discussion

Based on the inclusion and exclusion criteria established by the main MIS randomized trials published to date, implementation of MIS techniques for the treatment of ICH in a tertiary neurosurgical center of an European middle-income country would imply operating about 12.4% of ICH patients-per-year, if opting for the catheter-based technique, and around 43.0% of ICH patients-per-year, when opting for the neuroendoscopic evacuation. Overall, the balance of costs and benefits determined that MIS was a more expensive but more effective intervention than its conventional alternative and it could become a cost-effective strategy under a willingness-to-pay threshold of € 75,000–82,000. In our setting, the cost-utility analysis favored the endoscopic evacuation over the catheter plus rtPA procedure.

In our real-life model, the number of potential candidates for neuroendoscopic evacuation could triple that of potential candidates for the catheter technique. The outstanding difference in the number of prospective candidates between the two techniques was mainly due to the hematoma volume threshold, which was set at 30 ml in MISTIE III and at 10 ml in DIST. Another important divergence was the requisite of hematoma stabilization in MISTIE III, by which a second imaging was needed, separated at least 6 h from the first, showing no increase in the hematoma (or < 5 ml). In both groups, two frequent reasons for exclusion were a mRS of 3 or more and the presence of irreversible impaired brain stem function.

The cost-utility of the MIS strategy, compared to the conventional treatment, is determined by the willingness-to-pay threshold, which in turn is set by each local welfare system. Under the most generally accepted threshold in our local healthcare system (€ 30,000), MIS might not be cost-effective, neither in the catheter-based technique nor in the endoscopic version of it (21). Despite this consideration, the magnitude of effect size that MIS would need to achieve the threshold of € 30,000 per QALY would be higher for the catheter evacuation (0.72 QALY) than for the endoscopic evacuation (0.56 QALY). This could be deemed as a goal to achieve by the MIS techniques so as to become optimal for implementation.

Notwithstanding the effectiveness threshold, the cost-utility of MIS would change across healthcare systems depending on their cost acceptability limits. In fact, the American College of Cardiology and the American Heart Association recommend a cost-effectiveness level of $ 50,000 for high-value care and $ 50,000–15,000 for intermediate-value care (23). Under these thresholds, both the endoscopic and the catheter-based techniques would be qualified as intermediate-value care options. Overall, these discrepancies in healthcare policies highlight the value of the cost-utility acceptability curves. Of note, in the cost-utility graphics, the majority of the replicates for both MIS techniques fell in the north-east corner, which indicates a costlier and more effective intervention, when compared to the conventional assistance. Importantly, the cost of MIS is expected to decrease over time, as the devices entry secondary market competition and more affordable alternatives to the original procedures are conceived (24).

The volume of residual hematoma seems to be a paramount prognostic factor after MIS. In the future, to pursue better surgical results, an effort should be made to increase the rate of evacuation or the accuracy of the approach. Whether it is based on cautious patient selection (25), ultra-early evacuation (26), refinement of the surgical technique, association of neuroprotectives (27) or use of mechanical adjuvants to ease hematoma disaggregation, like sonothrombolysis (28) or transcranial histotripsy (29). Moreover, advances in the endoscopic ports have also been pursued to minimize white matter disruption (30), and robot assisted surgery has been developed to increase the accuracy in stereotactic placement of the catheter (31). All these comings outline a more sophisticated and potentially more cost-effective MIS intervention for ICH.

Ours is the first simulation of real-life implementation of the novel MIS techniques in the field of ICH. Comparison of our economic results can only be made with the study carried out by Vardanyan et al. (15), who performed a cost-utility analysis of the MISTIE III trial, according to the current costs of treatment in the NHS (United Kingdom). The authors concluded that the evaluated MIS technique did not meet the cost-utility threshold of € 30,000 to make it feasible for application in the NHS. This is in line with our own results; however, their estimated ICER exceeded ours by far. This could be attributed to either higher health-related costs in the UK, or the fact that theirs was not a real-life model but rather a direct comparison between the experimental and the control group of the MISTIE III trial.

One remarkable finding in our study was the fact that endoscopic evacuation seems to be more cost-effective than catheter aspiration, even when the cost of the endoscopic procedure clearly exceeds that of the catheter. The first randomized controlled study evaluating MIS for spontaneous supratentorial hematoma evacuation, MISTIE III, demonstrated safety of ICH evacuation and significant benefits in terms of survival (4, 9). *Post-hoc* studies have shown a correlation between the rate of hematoma evacuation and clinical outcomes. In particular, they demonstrated that end-of-treatment hematomas of < 15 ml had more favorable functional outcomes (13, 14). Evacuation rates seem superior with the endoscopic evacuation (32) than with the catheter (13), which could account for better functional outcomes in Kellner et al. trial than in MISTIE III. Eventually, this could explain why the endoscopic procedure, although being more expensive, may be more cost-effective. All in all, it is likely that professional experience and refinement of MIS techniques will lead to better incremental effectiveness rates in due course.

The main limitation of our study is the heterogeneity of the sources from which data were obtained. We used clinical-based information to elaborate the analysis because the aim was to create a real-life model. Still, the comparison groups could only be obtained from a randomized trial in the case of the catheter-based technique, given that no such data was available for the endoscopic technique at the time of the present analysis. Another relevant limitation is the inhomogeneous selection criteria chosen by the two randomized trials. Given that they are aimed for two different surgical techniques, these differences of criteria are somewhat understandable; however, they create selection bias in the comparison analysis. Adding to this idea, the fact that only 17 of our institutional patients were potential candidates for the catheter technique undermines the power of the analysis. In this line of thought, the fact that midterm functional status was not done exactly at the same time-point in each of the cohorts might influence our conclusions: MISTIE III reported outcomes at 1 year, Kellner et al. did so at 6 months, and our follow-up time was 3 months. Finally, we built a real-life model upon data from a single tertiary center and thus extrapolation of our results must be done with reservations.

Future studies should be based on data from randomized trials regarding endoscopic evacuation, as soon as they become available. Besides, multicentric studies would augment the power of such study, although heterogeneity in terms of hospital dynamics and costs must be taken into account. A clear consensus of inclusion and exclusion criteria for MIS in ICH is the ultimate aim; this would favor equality of treatment and would also facilitate combination of datasets for combined or comparable analysis.

## Conclusions

Provided that ongoing clinical trials are able to prove mortality and functional benefits from early surgical evacuation of spontaneous intracerebral hematomas, it might be reasonable to assume that a standardized MIS protocol for hemorrhagic stroke may soon be implemented in the public healthcare system. This highlights the need for accurate estimations of the number of patients that could benefit from this intervention and the corresponding logistics requirements. In a tertiary hospital, around one out of 10 ICH patients-per-year could be candidates for a catheter-based treatment, and around four in ten, for a neuroendoscopic intervention. Compared to the conventional treatment, MIS seems a more expensive and more effective treatment alternative. Particularly, among the two MIS variants, the endoscopic evacuation appears to be more cost-effective than the catheter plus rtPA based technique. Further studies are advisable as new data from ongoing randomized trials becomes available.

## Data Availability Statement

The raw data supporting the conclusions of this article will be made available by the authors, without undue reservation.

## Ethics Statement

The studies involving human participants were reviewed and approved by Clinical Research Ethics Committee from the Hospital Clínic de Barcelona (Reg. HCB/2021/1282). The patients/participants provided their written informed consent to participate in this study.

## Author Contributions

RT, SA, and AM: conception and design. JH, LR, AF, and AM: data collection. RT, SA, LP, LL, NdR, and AM: analysis and interpretation of data. AM and SA: drafting the article. RT, SA, LL, RM, NdR, and JE: revising it critically for important intellectual content. All authors listed have made a substantial, direct, and intellectual contribution to the work and approved it for publication.

## Conflict of Interest

The authors declare that the research was conducted in the absence of any commercial or financial relationships that could be construed as a potential conflict of interest.

## Publisher's Note

All claims expressed in this article are solely those of the authors and do not necessarily represent those of their affiliated organizations, or those of the publisher, the editors and the reviewers. Any product that may be evaluated in this article, or claim that may be made by its manufacturer, is not guaranteed or endorsed by the publisher.
