# Supplementary material for: Minimally Invasive Surgery for Spontaneous Intracerebral Hematoma. Real-Life Implementation Model and Economic Estimation
Source: Front Neurol. 2022 May 2;13:884157. doi: 10.3389/fneur.2022.884157 (PMC9108381; doi:10.3389/fneur.2022.884157)
Supplement: Supplementary file 1 [file Table_1.docx]

**Supplemental Table S1**. Inclusion and exclusion criteria from the main randomized clinical trials on minimally invasive surgical evacuation of supratentorial spontaneous hemorrhage. These were used as a reference for patient selection in the present real-life simulation model.

|  | MISTIE III | DIST | ENRICH | INVEST |
| --- | --- | --- | --- | --- |
| Inclusion criteria | - Spontaneous supratentorial ICH ≥ 30 mL, with a Glasgow Coma Scale (GCS) ≤ 14 or a NIHSS ≥ 6.  - Clot stability on CT scan done at least 6 hours after diagnostic CT (growth < 5 mL).  - Symptoms < 24 hours prior to diagnostic CT  - Ability to randomize between 12 and 72 hours after first CT.  - Systolic Blood Pressure < 180 mmHg sustained for 6 hours.  - Historical mRS 0 or 1.  - Age ≥ 18 | - Age ≥ 18  - NIHSS ≥ 2  - Supratentorial ICH confirmed by CT, without a CT-angiography confirmed causative vascular lesion  - Minimal lesion size 10 mL  - Intervention can be started within 8 hours from symptoms onset; or for controls presentation within 8 hours of symptom onset.  - Patient's or legal representative's written informed consent | - Age 18-80 years  - Pre-randomization head CT demonstrating an acute, spontaneous, primary ICH  - Manual ICH volume between 30 - 80 mL  - Study intervention can reasonably be initiated within 24 hours after the onset of stroke symptoms. If the actual time of onset is unclear, then the onset will be considered the time that the subject was last known to be well  - GCS 5 - 14  - Historical mRS 0 or 1 | - Age ≥ 22 and ≤ 80, or age < 85 with baseline mRS 0  - Supratentorial ICH of volume ≥ 30 mL < 80 ml  - CT/MR demonstrates ICH stability (< 5 cc growth) at 6 hours after admission scan  If the initial stability scan shows growth, a second stability scan can be performed q12h until stability is demonstrated or until eligibility for the study has lapsed.  - NIHSS ≥ 6  - GCS 5 - 15  - Historical mRS 0 to 2  - Symptom onset < 24 h prior initial CT  - Apollo MIES can be initiated within 72h of ictus/bleed  - SBP can be controlled < 160 mmHg and sustained at this level for at least 6 hours |
| Exclusion criteria | - Infratentorial hemorrhage.  - Intraventricular hemorrhage requiring treatment for mass effect or trapped ventricle. External ventricular drain to treat intracranial pressure is allowed.  - Thalamic bleeds with apparent midbrain extension with third nerve palsy or dilated and non-reactive pupils. Other (supranuclear) gaze abnormalities are not exclusions.  - Irreversible impaired brain stem function, GCS ≤ 4.  - Ruptured aneurysm, arteriovenous malformation (AVM), vascular anomaly, Moyamoya disease, hemorrhagic conversion of an ischemic infarct, recurrence of a recent (< 1 year) hemorrhage diagnosed with radiographic imaging.  - Unstable mass or evolving intracranial compartment syndrome.  - Platelet count < 100,000; international normalized ratio (INR) > 1.4.  - Any irreversible coagulopathy or known clotting disorder.  - Inability to sustain INR ≤ 1.4 using short- and long-active procoagulants  - Subjects requiring long-term anti-coagulation are excluded.  - Use of Dabigatran, Apixaban, and/or Rivaroxaban  - Internal bleeding, involving retroperitoneal sites, or the gastrointestinal, genitourinary, or respiratory tracts. Superficial or surface bleeding.  - Positive urine or serum pregnancy test  - Allergy/sensitivity to rt-PA.  - Prior enrollment in the study.  - Participation in a concurrent interventional medical investigation or clinical trial.  - Not expected to survive to the day 365 visit due to co-morbidities and/or are do not resuscitate/ do not intubate status  - Any concurrent serious illness that would interfere with the safety assessments  - Mechanical heart valve. Presence of bio-prosthetic valve(s) is permitted.  - Known risk for embolization, including history of left heart thrombus, mitral stenosis with atrial fibrillation, acute pericarditis, or subacute bacterial endocarditis.  - Any other condition that the investigator believes would pose a significant hazard to the subject if the investigational therapy were initiated.  Active drug or alcohol use or dependence that, in the opinion of the site investigator, would interfere with adherence to study requirements.  - In the investigator's opinion, the patient is unstable and would benefit from a specific intervention rather than supportive care plus or minus MIS+rt-PA removal of the ICH.  - Inability or unwillingness of subject or legal guardian/representative to give written informed consent. | - Pre-stroke disability, which interferes with the assessment of functional outcome at 90 days, i.e. mRS > 2  - Causative vascular lesion or other known underlying cause (e.g. tumor, cavernoma)  - Untreated coagulation abnormalities, including INR > 1.3 and treatment with oral thrombin or factor X antagonists; patients on vitamin K antagonist can be included after correction of the INR.  - Current known severe infection for which antibiotic treatment at time of ICH symptom onset  - Patient moribund (e.g. coning, bilateral dilated unresponsive pupils)  - Pregnancy | - Ruptured aneurysm, arteriovenous malformation (AVM), vascular anomaly, Moyamoya disease, venous sinus thrombosis, mass or tumor, hemorrhagic conversion of an ischemic infarct, recurrence of a recent (<1 year) ICH, as diagnosed with radiographic imaging  - NIHSS < 5  - Bilateral fixed dilated pupils  - Extensor motor posturing  - Intraventricular extension of the hemorrhage to involve >50% of either of the lateral ventricles  - Primary Thalamic ICH  - Infratentorial intraparenchymal hemorrhage including midbrain, pontine, or cerebellar  - Use of anticoagulants that cannot be rapidly reversed  - Evidence of active bleeding involving a retroperitoneal, gastrointestinal, genitourinary, or respiratory tract site  - Uncorrected coagulopathy or known clotting disorder  - Platelet count < 75,000, International Normalized Ratio (INR) > 1.4 after correction  - Patients requiring long-term anti-coagulation that needs to be initiated < 5 days from index ICH  - End stage renal disease  - Mechanical heart valve  - End-stage liver disease  - History of drug or alcohol use or dependence that, in the opinion of the site investigator, would interfere with adherence to study requirements  - Positive urine or serum pregnancy test  - Known life-expectancy of < 6 months  - No reasonable expectation of recovery, Do-Not-Resuscitate, or comfort measures only prior to randomization  - Participation in a concurrent interventional medical investigation or clinical trial.  - Inability or unwillingness of subject or legal guardian/representative to give written informed consent  - Homelessness or inability to meet follow up requirements | Imaging  - Expanding hemorrhage on stability CT/MR scan  "Spot sign" identified on CTA (May perform a second CTA at 12 hours to demonstrate resolution)  - Hemorrhagic lesion  - Hemorrhagic conversion of an underlying ischemic stroke  - Infratentorial hemorrhage  - Large associated intra-ventricular hemorrhage requiring treatment for IVH-related mass effect or shift due to trapped ventricle (EVD for ICP management is allowed)  - Midbrain extension/involvement  - Absolute contraindication to CTA, conventional angiography, and MRA  Coagulation Issues  - Absolute requirement for long-term anti-coagulation  - Known hereditary or acquired hemorrhagic diathesis, coagulation factor deficiency  - Platelet count < 100 x 103 cells/mm3 or known platelet dysfunction  - INR > 1.4, elevated prothrombin time or activated partial thromboplastin time (aPTT), which cannot be corrected or otherwise accounted for  Patient Factors  - GCS 3 or 4.  - High risk condition for ischemic stroke  - Requirement for emergent surgical decompression or uncontrolled ICP after EVD  - Unable to obtain consent from patient or appropriate surrogate  - Pregnancy, breast-feeding, or positive pregnancy test  - Evidence of active infection  - Any comorbid disease or condition expected to compromise survival or ability to complete follow-up assessments through 180 days.  - Based on investigator's judgment, patient does not have the necessary mental capacity to participate or is unwilling or unable to comply with protocol follow up appointment schedule.  - Active drug or alcohol use or dependence that, in the opinion of the site investigator would interfere with adherence to study requirements.  - Currently participating in another interventional (drug, device, etc) research project. |
