# Supplementary material for: Minimally Invasive Surgery for Spontaneous Intracerebral Hematoma. Real-Life Implementation Model and Economic Estimation
Source: Front Neurol. 2022 May 2;13:884157. doi: 10.3389/fneur.2022.884157 (PMC9108381; doi:10.3389/fneur.2022.884157)
Supplement: Supplementary file 2 [file Table_2.docx]

**Supplementary Table S2**. Utility values for each score of the modified Rankin Scale (mRS) converted to Quality of Life Years (QALY).

| **mRS** | **QALY** |
| --- | --- |
| 0 | 0.93 |
| 1 | 0.85 |
| 2 | 0.71 |
| 3 | 0.55 |
| 4 | 0.28 |
| 5 | -0.15 |
| 6 | 0 |
